# Noncoding RNAs: the crucial role of programmed cell death in osteoporosis

**DOI:** 10.3389/fcell.2024.1409662

**Published:** 2024-05-10

**Authors:** Juanjuan Han, Yuqing Zhu, Jiale Zhang, Leonid Kapilevich, Xin-an Zhang

**Affiliations:** ^1^ College of Exercise and Health, Shenyang Sport University, Shenyang, China; ^2^ Faculty of Physical Education, Tomsk Stаte University, Tomsk, Russia

**Keywords:** noncoding RNA, programmed cell death, osteoporosis, osteoblast, osteoclast

## Abstract

Osteoporosis is the most common skeletal disease characterized by an imbalance between bone resorption and bone remodeling. Osteoporosis can lead to bone loss and bone microstructural deterioration. This increases the risk of bone fragility and fracture, severely reducing patients’ mobility and quality of life. However, the specific molecular mechanisms involved in the development of osteoporosis remain unclear. Increasing evidence suggests that multiple noncoding RNAs show differential expression in the osteoporosis state. Meanwhile, noncoding RNAs have been associated with an increased risk of osteoporosis and fracture. Noncoding RNAs are an important class of factors at the level of gene regulation and are mainly involved in cell proliferation, cell differentiation, and cell death. Programmed cell death is a genetically-regulated form of cell death involved in regulating the homeostasis of the internal environment. Noncoding RNA plays an important role in the programmed cell death process. The exploration of the noncoding RNA-programmed cell death axis has become an interesting area of research and has been shown to play a role in many diseases such as osteoporosis. In this review, we summarize the latest findings on the mechanism of noncoding RNA-mediated programmed cell death on bone homeostasis imbalance leading to osteoporosis. And we provide a deeper understanding of the role played by the noncoding RNA-programmed cell death axis at the gene regulatory level of osteoporosis. We hope to provide a unique opportunity to develop novel diagnostic and therapeutic approaches for osteoporosis.

## 1 Introduction

Osteoporosis (OP) is a progressive bone disease characterized by bone mass loss and microstructure deterioration, increasing the risk of fragility fractures (Akkus and Gulsen-Atalay, 2020). Factors like genetics, environment, estrogen deficiency, and medication contribute to OP. It results from osteoclast-mediated bone resorption exceeding osteoblast-mediated bone formation (Dell'Aquila et al., 2020). OP is becoming increasingly common in women after the age of 55 and in men after the age of 65, resulting in a high number of fractures and bone-related complications, increased mortality, and healthcare costs. Currently, the test for OP is the bone mineral density (BMD) score, but this lacks sensitivity making early diagnosis difficult. Meanwhile, the available treatments are imperfect. Understanding OP pathophysiology is crucial for early diagnosis and treatment.

Noncoding RNAs (ncRNAs) are a class of RNA molecules that cannot code for proteins. This type of RNA mainly includes microRNAs (miRNAs), long non-coding RNAs (lncRNAs), and circular RNAs (circRNAs). NcRNAs are crucial contributors to diseases related to cell proliferation, cell differentiation, and cell death (Slack and Chinnaiyan, 2019). It can affect the occurrence and development of OP by regulating the expression of target genes and epigenetics (Terashima et al., 2017). Recent studies have also found that some ncRNAs can be translated to generate peptides or small proteins. Some ncRNAs can encode functional peptides through their small open reading frames, which are involved in cellular signaling, regulation of gene expression, and other biological processes, and thus affect bone cell Functions of osteoblasts (Guan et al., 2022). Programmed cell death (PCD) is vital for tissue development and homeostasis, but its imbalance leads to diseases. Recent studies have shown that ncRNAs are widely associated with the regulation of PCD, and the association of the ncRNAs-PCD axis with cancer has become one of the hottest topics in biomedical sciences. In recent years, numerous studies have found that the ncRNAs-PCD axis is closely related to OP (Behera et al., 2022; Zhao et al., 2024). OP can be viewed as an imbalance of PCD in osteocytes, and similarly, the ncRNAs-PCD axis plays a role in bone metabolism and bone formation. In this paper, we review and explore the role of ncRNAs in OP, focusing on their interplay with PCD, aiming to identify new treatment targets and guide clinical interventions for OP.

## 2 Noncoding RNAs and osteoporosis

### 2.1 Noncoding RNAs

**FIGURE 1:**
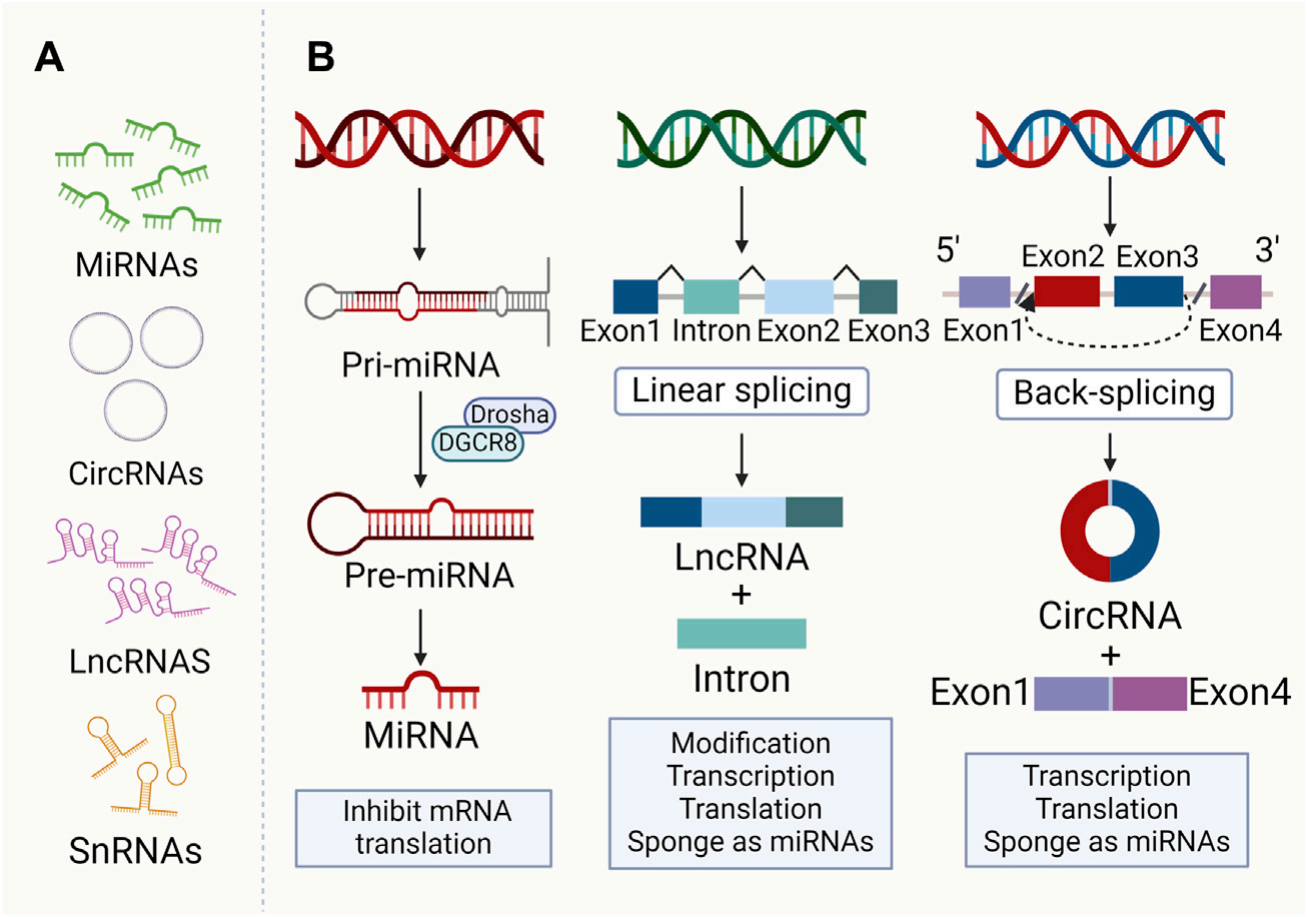
The morphology and formation of ncRNAs. **(A)** Morphology of main ncRNAs: MiRNAs are 18–25 nt. CircRNAs are 100–10,000 nt with a closed loop. LncRNAs are over 200 nt. SncRNAs consist of various small RNAs. **(B)** Major ncRNA formation and function. Pri-miRNAs are converted to pre-miRNAs by Drosha and DGCR8/Pasha, then matured by Dicer. LncRNAs result from the linkage of multiple small RNAs. CircRNAs are generated through a back-splicing of 3′ and 5′ RNA ends.

NcRNAs are RNA molecules produced during genome transcription that do not encode proteins. They include small molecules like miRNA, small interference RNA (siRNA), circRNA, small non-coding RNAs (sncRNA), and larger molecular weight ones like lncRNA (St Laurent et al., 2015). These RNAs regulate cell processes such as proliferation, differentiation, and death (Slack and Chinnaiyan, 2019). LncRNAs, over 200 nt, regulate gene expression and cellular processes, impacting protein translation and stability (Zhang et al., 2017b). They can also act as decoys, scaffolds, competing endogenous RNAs (ceRNAs), etc., leading to disease ultimately (Thomson and Dinger, 2016). MiRNAs, 18-25 nucleotides long, regulate post-transcriptional gene expression (Lu and Rothenberg, 2018). MiRNAs in their most primitive form are primary miRNAs (pri-miRNAs). Pri-miRNAs are processed by RNA endonuclease III (Drosha) and DGCR8/Pasha to become pre-miRNAs, a microRNA precursor. The pre-miRNA is then cleaved by the enzyme Dicer and becomes a mature miRNA. MiRNAs control almost all pathways and directly control cell proliferation, differentiation, morphogenesis, and apoptosis. Therefore, they are essential for maintaining or determining cell survival (Cai et al., 2009). CircRNAs are ncRNAs with a covalent closed-loop structure, ranging from about one hundred to several thousand nucleotides, and are produced by 3′ to 5′ end-joining events (reverse splicing) (Ebbesen et al., 2017). CircRNAs not only act as miRNA sponges or ceRNAs (Tay et al., 2014), which compete with other RNAs for miRNA pairing, but regulate transcription in the nucleus and bind to protein factors (Ashwal-Fluss et al., 2014) (as shown in Figure 1). SncRNAs consist of various RNA types, including tRNA-derived small RNAs, small nucleolar RNAs (snoRNAs), small nuclear RNAs (snRNAs), PIWI-interacting RNAs, etc (Xiao et al., 2022). At present, studies on snRNA and snoRNA mainly focus on tumors rather than osteoporosis, presenting a potential new research avenue.

### 2.2 The regulation of noncoding RNAs in osteoporosis

**FIGURE 2:**
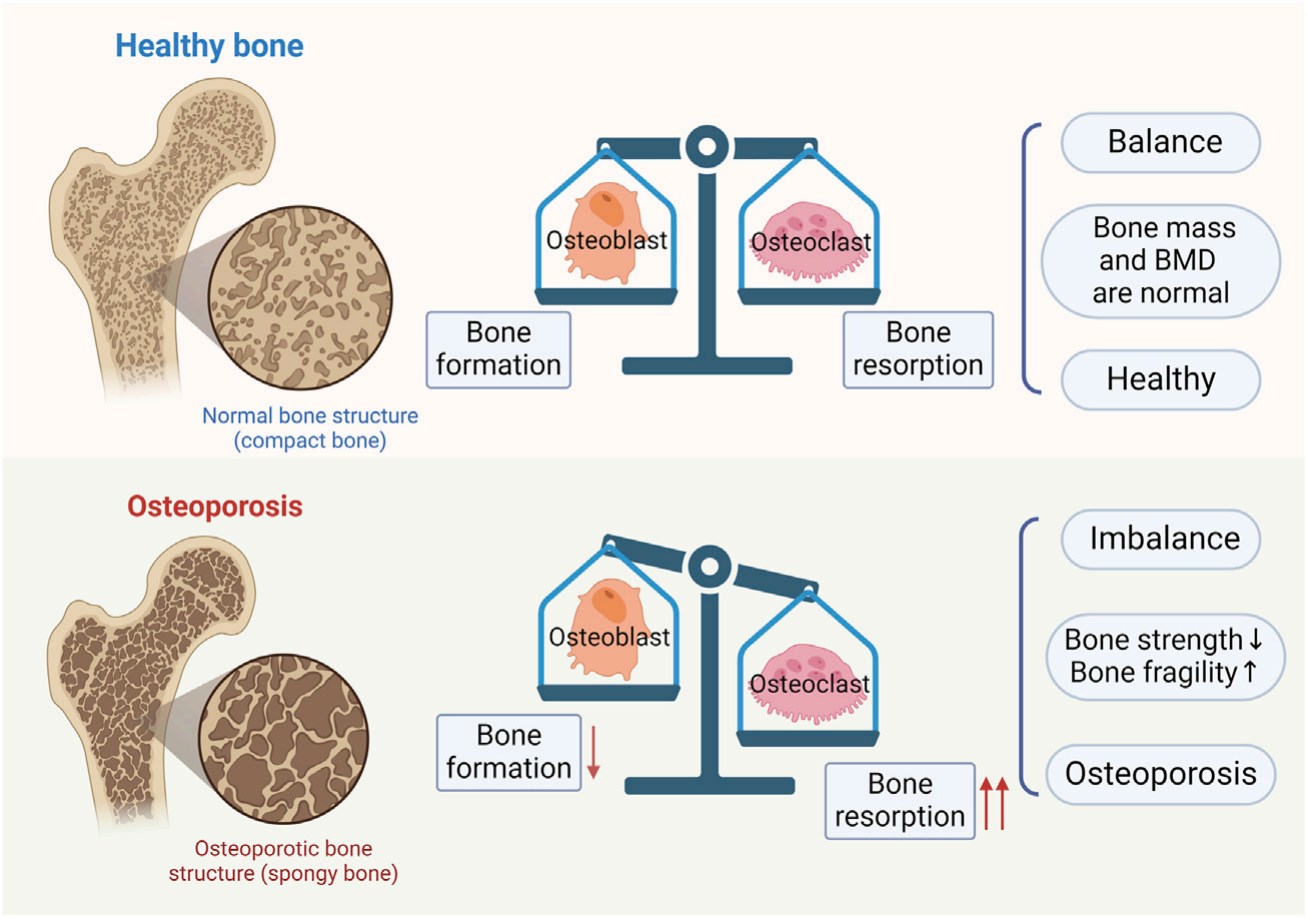
Bone homeostasis and osteoporosis. In normal bones, bone formation by osteoblasts and bone resorption by osteoclasts are in balance. When there is an imbalance between bone formation by osteoblasts and bone resorption by osteoclasts, osteoporosis results.

Osteoblasts are involved in bone formation through a variety of pathways, including the synthesis and secretion of bone matrix, promotion of mineral deposition, repair of bone and formation of new bone, and secretion of growth factors and cytokines for the maintenance of bone mass and density. Osteoclasts and osteoblasts have opposite functions, including resorption or dissolution of bone tissue, inhibition of mineral deposition, removal of old bone and damage to bone tissue (Kim et al., 2020). Their balance ensures proper repair and renewal of bone tissue, keeping bone mass and BMD within normal limits. However, when osteoblasts are inhibited or osteoclasts are over-activated, the balance of bone resorption and bone formation is disrupted, leading to OP (as shown in Figure 2).

NcRNAs play a key role in bone metabolism, and OP leads to abnormal expression of certain ncRNAs *in vivo*. Not only that, gene regulation and gene modification related to bone metabolism also affect ncRNA expression. OP also leads to abnormalities in some signaling pathways, resulting in activation or inhibition of ncRNA expression (Terashima et al., 2017). LncRNAs improve OP by promoting the osteogenic differentiation of bone marrow mesenchymal stem cells (BMSCs) and adipose mesenchymal stem cells and inhibiting their adipogenic differentiation (Liu et al., 2021b). They participate in OP by promoting osteoblast differentiation, inhibiting osteoclast proliferation, and altering the expression of target genes (Zhang et al., 2019). MiRNAs can likewise be involved in the regulation of bone metabolism, by regulating osteogenic and osteoclastic differentiation. MiRNAs target transcription factors and signaling molecules of osteoblast production and osteogenic function pathway and affect other substances related to osteoclastic differentiation (Kobayashi et al., 2016). Several miRNAs (miR-124, miR-193-3p) regulate osteoclast differentiation by targeting osteoclast-associated mRNAs (Li et al., 2019; Ohnuma et al., 2019). Dysfunction of miRNAs disrupts bone remodeling by reducing bone anabolic function in osteoblasts (Lei et al., 2011). In bone metabolism, on the one hand, circRNA regulates stem cell osteogenic differentiation and monocyte macrophage osteoclast differentiation. On the other hand, circRNA regulates various osteoblast signaling pathways, such as Wnt and RANKL-RANK pathway. It can also serve as a biomarker for OP, acting as a ceRNA for miRNAs involved in bone metabolism. Nowadays, existing studies have found that snRNA and snoRNA have important roles in cancer, immunity, amyotrophy, and osteoarthritis (Bisht et al., 2020). SnoRNA affects chondrogenic and hypertrophic gene expression when its expression is altered (Peffers et al., 2020). However, the role of snRNA and snoRNA in OP has not been reported, so they may be a promising research direction.

## 3 Programmed cell death and osteoporosis

### 3.1 Programmed cell death

**FIGURE 3:**
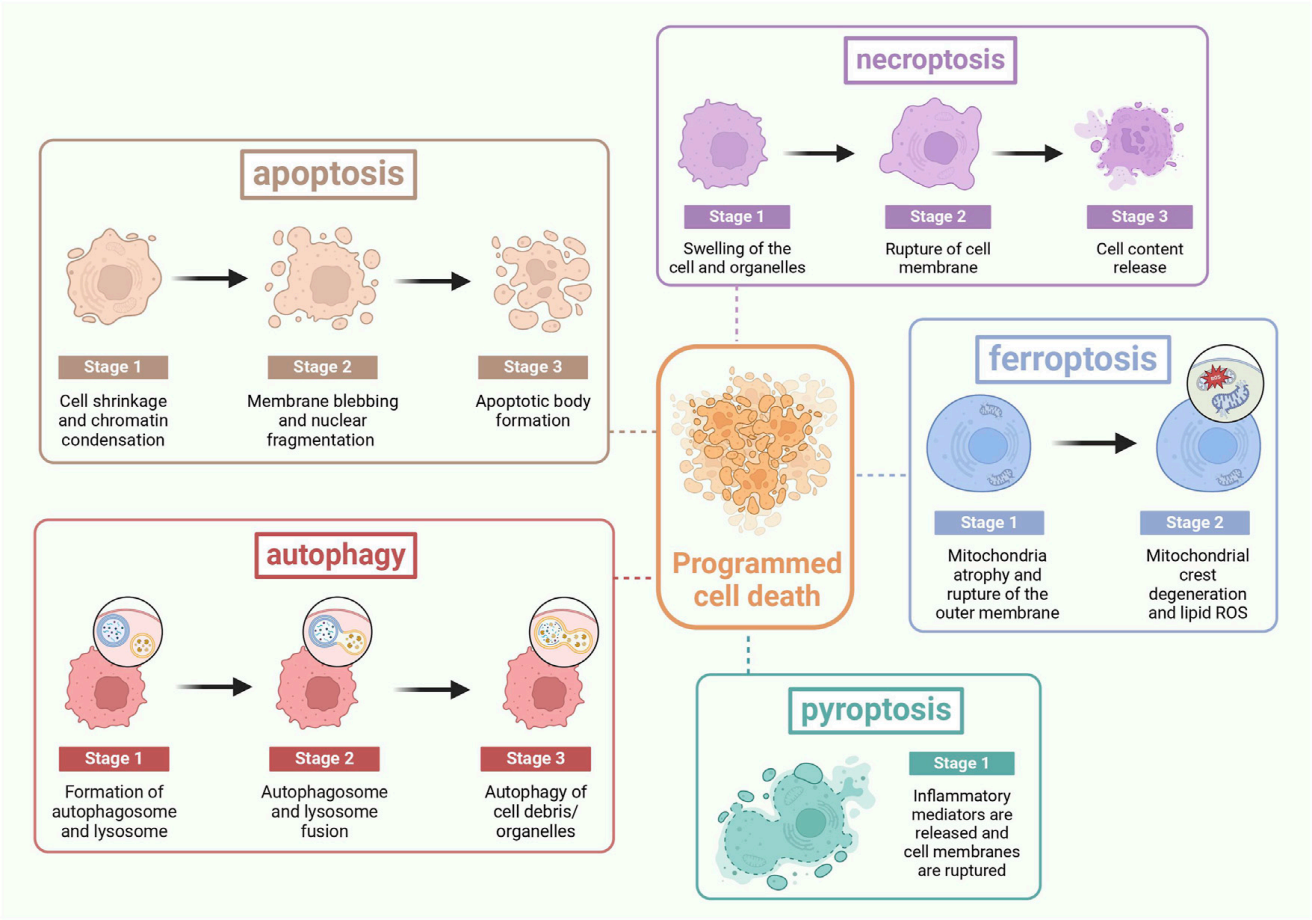
The process of PCD: Apoptosis is characterized by cell shrinkage, membrane blebbing, and the formation of apoptotic bodies. Necroptosis is marked by cell swelling, rupture of the cell membrane, and cell lysis. Ferroptosis features the rupture of the cell membrane, cytoplasmic swelling, mitochondrial atrophy, and the alteration of lipid reactive oxygen species. Pyroptosis is identified by the rupture of the cell membrane and the release of inflammatory mediators. Autophagy is characterized by the fusion of autophagosomes with lysosomes. PANoptosis is distinguished by the release of inflammatory mediators and the rupture of the cell membrane.

PCD, a genetically controlled cell death process, is crucial for maintaining internal balance and can lead to various diseases if imbalanced (Tsuda et al., 2012). PCD has several manifestations (as shown in Figure 3). Apoptosis is characterized by membrane blister, apoptotic body production, nuclear condensation and organelle/DNA fragmentation. These alterations ultimately lead to cellular disintegration, which is phagocytosed by phagocytic housekeepers from innate immunity without releasing pro-inflammatory cellular contents into the extracellular milieu (Liu et al., 2022a). Apoptosis is involved in many physiological mechanisms such as maintenance of tissue differentiation, organ development, and aging, damaging and eliminating body hemostasis in mutated cells (Miura et al., 1993). Necroptosis was described as a novel, TNF-α-triggered form of non-apoptotic cell death that occurs in the absence of caspase-8 (Degterev et al., 2005). Necroptosis has long been recognized as a passive and unregulated process in response to severe pathological stress, characterized by cellular swelling, destruction of cell membranes and organelles, and cell lysis (Edinger and Thompson, 2004). It is a cellular response to environmental stress, which may be induced by chemical and mechanical injury, inflammation, or infection (Khoury et al., 2020). Ferroptosis results from iron-dependent lipid peroxidation (Hu et al., 2022). The cells typically exhibit necrotic-like morphologic transformations, including rupture of cellular membranes, cytoplasmic swelling, and as well as mitochondrial atrophy, and lipid reactive oxygen species (ROS) (Tang et al., 2021). It has two main pathways, the extrinsic or transporter protein-dependent pathway and the intrinsic or enzyme-regulated pathway. Pyroptosis is an active and organized inflammatory form (Li et al., 2021). It is usually caused by microbial infection, accompanied by activation of the inflammatory vesicles and maturation of the pro-inflammatory cytokines IL-1β and IL-18 (Tan et al., 2021b). It has two signaling pathways: a canonical pathway mediated by cysteine-1 and a non-canonical pathway mediated by cysteine-11 (Rühl et al., 2018). It is characterized by the activation of inflammatory cysteine-1 enzymes and cleavage of various members of the gasdermin family to form membrane-perforating, leading to cell membrane rupture, the release of inflammatory mediators and cell death ultimately (Wei et al., 2022). Autophagy refers to the catabolic process by which a cell turns over its own components (Yang and Klionsky, 2010). It is an important fundamental metabolic mechanism that affects cell survival by maintaining cellular bioenergy and removing protein aggregates and damaged organelles (Doherty and Baehrecke, 2018). During autophagy, cytoplasmic materials and organelles are segregated and phagocytized by double-membrane structures called autophagosomes and transported to lysosomes for degradation and recycling (Ichimiya et al., 2020). PANoptosis combines features of pyroptosis, apoptosis, and necroptosis in a coordinated pathway, triggered by various factors like infection or injury, with critical PAN photoreceptor assembly and activation (Pandian and Kanneganti, 2022).

### 3.2 The regulation of programmed cell death in osteoporosis

Adult bone mass is regulated by the balance between osteoclast and osteoblast production and their apoptosis (Weinstein and Manolagas, 2000). Approximately 60%–80% of osteoblasts die from apoptosis (Jilka et al., 2007), a process influenced by factors like aging, sex steroid deficiency, and glucocorticoid(GC) overload, leading to bone loss and OP. GC-induced OP is due to the activation of cysteinyl asparaginase 3, a general downstream effector of the apoptosis signaling pathway, which induces apoptosis in osteoblasts (Li et al., 2012). Estrogen can attenuate OP by attenuating apoptosis in osteoblasts through Fas gene expression. Thus, pharmacologic inhibition of osteoclast apoptosis may prevent the activation of bone resorption and bone loss. And it could be further explored as a direction for clinical research. Studies have shown that inhibition of necroptosis always contributes to bone formation. Necroptosis inhibitor (Nec-1) can regulate RIPK1/RIPK3/MLKL axis, leading to osteoblast necroptosis decrease, and amelioration of OP (Feng et al., 2014; Han et al., 2018). Subsequently, Guo et al. showed that excessive ethanol intake in mice can result in increased osteoblast necroptosis and decreased bone formation (Guo et al., 2021). In addition, in ovariectomized (OVX) rats, when E2 is deficient, there is an increase in necroptosis of osteoblasts (Cui et al., 2016). Ferroptosis is an iron-dependent form, and the amount of iron in the body’s environment can influence the expression of bone cell ferroptosis. Large accumulations of ROS in the skeleton trigger lipid peroxidation leading to bone cell ferroptosis (Stockwell et al., 2017). There is a positive correlation between the OP and the severity of iron overload (Tsay et al., 2010). Inhibiting osteoblast ferroptosis can enhance osteoblast differentiation and mineralization capacity. For instance, hepcidin increases can reduce ROS-induced lipid peroxidation and activate the BMP-2/Smad pathway, leading to a decrease in osteoblast ferroptosis and promoting osteogenic differentiation of BMSC (Lu et al., 2015; Jiang et al., 2019). The promotion of osteoclast ferroptosis is beneficial for OP. Osteoclasts are abundant in mitochondria, so when the iron transfer protein receptor TfR1 increases and the iron supply to mitochondrial respiratory protein increases, it activates osteoclast ferroptosis and relieves OP (Ishii et al., 2009). Pyroptosis may lead to functional inhibition of osteoblasts, and activation of osteoclasts (Wang et al., 2022b). It leads to death or functional inhibition of osteoblasts, as well as excessive proliferation and activation of osteoclasts. Oxidative stress induced by hypoxia, hyperglycemia, lipopolysaccharide and ATP stimulation can increase osteoblast pyroptosis (Liu et al., 2020a). Inflammatory vesicles such as IL-1β, TNF-α, and NLRP3 can trigger osteoclast pyroptosis, further contributing to bone resorption and OP improvement (Dai et al., 2004; Qu et al., 2015). Autophagy imbalance can result in bone loss. It is essential for the biological functions of BMSCs, osteoblasts, and osteoclasts. Activation of autophagy enables BMSCs to survive under stressful conditions such as nutrient deficiency, oxidative stress, and inflammation (Yang et al., 2016). It provides the energy for osteogenic differentiation and bone metabolism remodeling, protecting osteoblasts from death when exposed to oxidative stress, inflammation, and GCs (Zheng et al., 2020b). The differentiation, survival, migration, and bone resorption of osteoclasts also rely on autophagy. Disruption of autophagy leads to changes in osteoclast function, increased bone loss, and ultimately contributes to the development of OP. PANoptosis, a combined form of pyroptosis, apoptosis, and necroptosis (Zhu et al., 2023), is less studied in relation to OP but may offer new insights in future research.

## 4 The interaction between noncoding RNAs and programmed cell death

### 4.1 Noncoding RNAs and apoptosis

**FIGURE 4:**
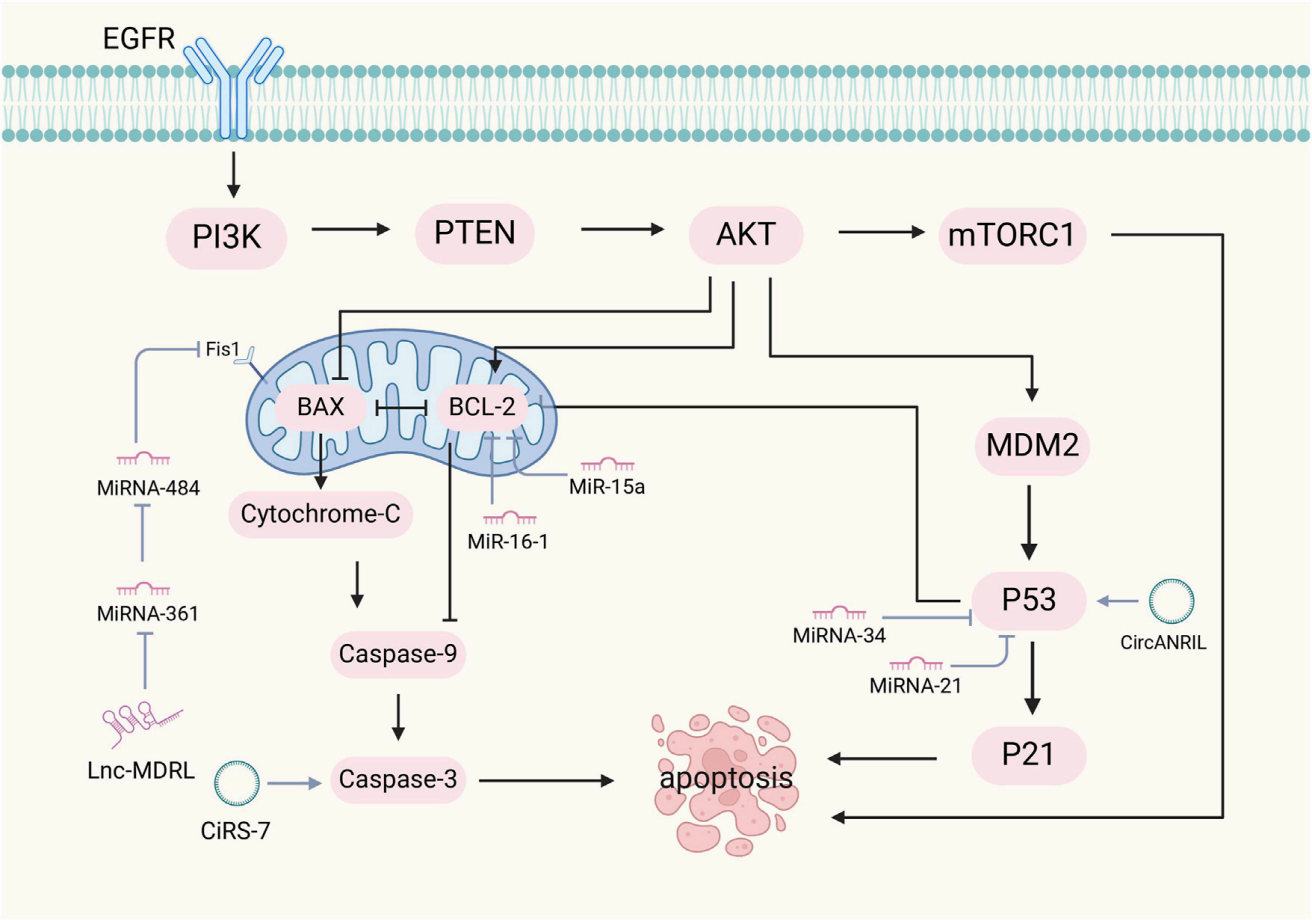
Mechanism of ncRNA regulation of apoptosis. The mechanisms by which ncRNAs regulate apoptosis include the p53-dependent pathway, mitochondrial dynamics, and the EGFR/PI3K/PTEN/AKT/mTORC1 signaling pathway. NcRNAs with the p53-dependent apoptotic pathway are affected by caspases and BCL-2 family proteins. Caspases activate upon receipt of apoptotic signals, leading to cytoplasmic and nuclear protein cleavage, triggering DNA enzyme activation and DNA fragmentation. BCL-2 proteins inhibit pro-apoptotic proteins and maintain mitochondrial membrane integrity.

The regulation of apoptosis by ncRNAs, particularly lncRNAs, is crucial for disease prevention. LncRNAs influence apoptosis through p53-dependent pathways, mitochondrial dynamics, and the EGFR/PI3K/PTEN/AKT/mTORC1 signaling cascade. The interaction between lncRNAs and p53-dependent apoptosis was affected by caspases and pro/antiapoptotic proteins. Caspase is divided into two groups named initiator and executioner caspases. When cells receive proapoptotic signals, initiator caspases are activated, triggering executor procaspases to cleave specific cytoplasmic and nuclear proteins as apoptosis markers. DNA enzymes are activated, genomic DNA is fragmented, and apoptosis occurs (Boatright and Salvesen, 2003). Apoptosis is also regulated by the BCL-2 family of proteins. They act as anti-apoptotic proteins that inhibit the activity of proapoptotic effectors (BAK and BAX), leading to the disruption of the outer mitochondrial membrane. Upon damage to the mitochondrial outer membrane, cytochrome c and ATF endonuclease are released. Cytochrome c binds to apoptosis protease activating factor-1 and forms apoptotic bodies, which activate caspase-9. Caspase-9 then activates caspase-3, caspase-6, and caspase-7, which contribute to the cleavage of other proteins and ultimately lead to apoptosis (Wolf et al., 2022). Mitochondrial dynamics play a crucial role in apoptosis, with abnormal dynamics contributing to disease pathogenesis (Scott and Youle, 2010). Abnormal mitochondrial dynamics impair apoptosis and contribute to a variety of diseases. Inhibition of the mitochondrial fission machinery prevents apoptosis. Different lncRNAs have different roles in mitochondrial fission. MDRL inhibits mitochondrial fission by miR484 induction and fission 1(Fis1) inhibition. CARL also induces mitochondrial fission inhibition by inhibiting miR593 and then activating Prohibitin-2. However, inhibiting the MPRL of miR483 guides mitochondrial fission by Fis1 upregulation (Asl et al., 2022). In addition, mitochondrial fusion was inhibited during apoptosis (Suen et al., 2008). Outer membrane fusion is mediated by OPA1, while inner membrane fusion is regulated by mitochondrial fusion protein (MFN)1 and two proteins. Pro-apoptotic proteins inhibit mitochondrial fusion by forming complexes with MFN1 and two proteins, leading to apoptosis induction (Suen et al., 2008). The EGFR/PI3K/PTEN/AKT/mTORC1 pathway, an important signaling pathway involved in mammalian metabolism, regulates lncRNAs. Various extracellular ligands such as epithelial growth factor (EGF) can bind to receptor tyrosine kinases (RTKs) cell surface receptor families, such as EGFR. It initiates the process of autophosphorylation in RTK, resulting in phosphoinositide 3-kinase (PI3K) activation. The PI3K enzyme converts the cell membrane protein called phosphatidylinositol 4,5-bisphosphate to phosphatidylinositol (3,4,5) -trisphosphate, thereby activating protein kinase B (AKT), which is necessary in cell metabolism (Noorolyai et al., 2019). AKT initiates protein synthesis, which leads to apoptosis inhibition (Zhang et al., 2017a). Dysregulation of this pathway can lead to disease due to abnormal cell cycle activity (As shown in Figure 4).

MiRNAs primarily function as inhibitors of apoptosis, modulating this process through p53 and TGF-β signaling (Babashah and Soleimani, 2011). MiR-21 associates with p53 which can inhibit apoptosis. It is manifested by miR-21 targeting the p53 network, TGF-β and mitochondrial apoptosis inhibitory genes in transformed cells (Papagiannakopoulos et al., 2008). Chen et al. found that lowering miR-21 triggered the activation of cysteine asparaginase and led to increased apoptosis (Chan et al., 2005). Similarly, de-repression of the p53 pathway by miR-21 downregulation may contribute to the inhibition of the cytostatic response to TGF-β signaling, leading to increased apoptosis. The miR-34 family, a p53 effector, regulates the reprogramming involved in p53-mediated apoptosis (Mayr et al., 2007), thereby allowing p53 to maintain tissue homeostasis by promoting cell cycle arrest and apoptosis in response to stress signals (Fridman and Lowe, 2003). Additionally, miRNAs like MiR-15a and MiR-16-1 interact with the anti-apoptotic BCL2, leading to cleavage of procaspase 9 and poly ADP-ribose polymerase, which results in activation of the intrinsic apoptosis pathway and promotes apoptosis (Lynam-Lennon et al., 2009). Mcl-1 is a member of the Bcl-2 family of potent multidomain anti-apoptotic proteins that can bind to the pro-apoptotic members Bim and Bid (Chen et al., 2005). MiR-29 expression enhancement can reduce Mcl-1 protein levels to promote apoptosis (Mott et al., 2007).

CircRNA regulate apoptosis through circRNA-miRNA-mRNA networks. CircANRIL triggers apoptosis by directly activating p53 and by impairing rRNA biogenesis (Holdt et al., 2016). In contrast, circ_0005105 and circRNA.33186 promotes chondrocyte apoptosis by activating the miR-26a/NAMP axis and negatively regulating miR-127-5p (Zhou et al., 2019). CircRNA_Atp9b facilitates apoptosis in osteoarthritis mouse models by negatively regulating miR-138-5p (Zhou et al., 2018a).

### 4.2 Noncoding RNAs and ferroptosis

Ferroptosis is regulated by ncRNAs, which modulate mitochondrial proteins, iron metabolism, glutathione (GSH), and lipid peroxidation (Wang et al., 2020b). MiRNAs targeting iron metabolism are categorized into ferritin-resistant and ferritin-promoting types. When the levels of miRNAs are altered, the organism may undergo iron deficiency, leading to cellular failure, and iron overload, resulting in increased cellular oxidative stress and increased ferroptosis. Ferritin-resistant type miRNA, such as miR-7-5p, reduces iron uptake, targets transferrin, and indirectly reduces unstable iron pools and Fenton response. Ferritin-promoting type miRNA can either directly target the SLC7A11/GPX4 system to promote lipid peroxidation or, like miR-214, be regulated by the ATF4 target gene HSPA5, which indirectly leads to ferroptosis (Fuhrmann and Brüne, 2022). MiRNAs affect antioxidant metabolism by targeting the SLC7A11/GPX4 system. MiRNAs inhibit GPX4 activity by inhibiting SLC7A11, preventing cystine from entering the cell, and reducing the supply of GSH. Impairment of the antioxidant system of the cell leads to the inability to remove lipid peroxides in time and ferroptosis occurs (Dai et al., 2022). For instance, miR-5096, by inhibiting the activity of SLC7A11, leads to an increase in iron content, ROS, hydroxyl radicals, lipid peroxides, and a decrease in GSH, and ultimately induces ferroptosis in breast cancer cells (Yadav et al., 2021). MiR-15a-3p directly binds to the 3′-UTR of GPX4 and inhibits its activity, leading to an increase in intracellular ROS, Fe2+ levels, and ultimately to ferroptosis in colorectal cancer cells (Liu et al., 2022b). MiRNAs also regulate ferroptosis by affecting lipid metabolism. During ferroptosis, arachidonic acid is the target of intracellular ROS attack, and its diallyl C-H is susceptible, leading to lipid peroxidation and PCD (Yin et al., 2011). MiR-522 prevents cellular lipid peroxidation by inhibiting arachidonic acid lipoxygenase 15 activity to prevent lipid peroxidation in cells, thereby inhibiting ferroptosis (Zhang et al., 2020a).

LncRNAs serve as miRNA sponges to modulate ferroptosis. For example, MT1DP in NSCLC cells enhances ferroptosis by stabilizing miR-365a-3p and reducing NRF2 (Gai et al., 2020). RP11-89 sponges miR-129-5p, promoting PROM2-mediated ferritin-containing multivesicular bodies formation and iron release, which enhances ferroptosis resistance (Luo et al., 2021). LncRNA OIP5-AS1 plays a role in inhibiting prostate cancer cells ferroptosis by regulating the miR-128-3p/SLC7A11 axis (Zhang et al., 2021c). LncRNA can act alone to regulate ferroptosis. High expression of LINC00336 inhibits ferroptosis by decreasing intracellular iron and lipid ROS levels (Wang and Tontonoz, 2019). LINC00618 inhibits SLC7A11 expression by directly interacting with lymphoid-specific helicase, resulting in increased ferroptosis (Wang et al., 2021f). CircRNAs are involved in ferroptosis in a manner similar to lncRNAs. Some circRNAs can act as miRNA sponges targeting SLC7A11 as well as other ligands to inhibit ferroptosis (Xian et al., 2020; Wu et al., 2021). There are also circRNAs that act individually such as CIARS, which regulate ferroptosis by interacting with formed proteins (Liu et al., 2020b).

### 4.3 Noncoding RNAs and autophagy

Autophagy plays a crucial role in disease development and drug resistance by targeting autophagy-related proteins and forming competitive networks. Autophagy-related lncRNAs can either activate or inhibit autophagy by interacting with DNA, RNA, or proteins (Cech and Steitz, 2014), or by regulating ATG expression and acting as ceRNAs for miRNAs. The lncRNA NBR2 facilitates autophagy initiation under energetic stress by directly activating AMPK, which is beneficial for ameliorating disease (Liu et al., 2016). However, activation of autophagy may also impair cell viability. Activation of ERK1/2, a protein kinase phosphatase for autophagy initiation, triggers autophagy. LncRNA H19 activates autophagy by inhibiting DUSP5, which diminishes the inhibitory effect of DUSP5 on ERK1/2, induces cerebral ischemia reperfusion injury (Wang et al., 2017). Sometimes, lncRNAs can act together with miRNAs to regulate autophagy. Overexpression of maternally expressed gene 3 (MEG 3), a lncRNA with tumor-suppressor function, inhibits the development of epithelial ovarian cancer by inducing the autophagy pathway through upregulation of Atg3 activity and protection of its mRNA from degradation (Xiu et al., 2017). In addition, GAS5 is highly expressed lncRNA in OA and promotes the pathogenesis of OA by acting as a negative regulator of miR-21, which may inhibit autophagy by down-regulating Beclin 1, ATG3, ATG5, ATG12, and LC3B expression (Song et al., 2014). MiRNAs also play a significant role in autophagy regulation by targeting autophagy-related proteins or signaling pathways. MiR-30a inhibits autophagy in chronic myeloid leukemia and hepatocellular carcinoma (HCC) cells by selectively blocking BECN1 and ATG5, which are needed for autophagy nucleation and elongation stages respectively (Yu et al., 2012; Fu et al., 2018b). MiR-101 could act as an inhibitor of autophagy in HCC through RAB5A, ATG4D and Stathmin1 targeting (Xu et al., 2013). Meanwhile, miRNAs also target signaling pathways to regulate autophagy. MiR-22 reduces chemoresistance in osteosarcoma cells by inhibiting autophagy through inactivation of the PI3K/Akt/mTOR pathway (Meng et al., 2020). In melanoma cells, miR-23a overexpression inhibits autophagy by targeting ATG12 via the AMPK-RhoA pathway (Guo et al., 2017). CircRNAs regulate autophagy by acting as miRNA sponges or binding to RNA binding proteins (Zhou et al., 2022a). Binding of circRNAs to specific miRNAs is a key pathway that affects the autophagy process, such as CircEEF2 and miR-6881-3p (Yong et al., 2020), circCDYL and miR-1275 (Liang et al., 2020), circMTO1 and miR-6893 (Chen et al., 2019). The circRNA-miRNA networks mentioned above can all promote autophagy. However, some networks inhibit autophagy. Zhou et al. demonstrated that circRNA.2837 can inhibit autophagy by acting as a sponge for miR-34a (Zhou et al., 2018b). CircRNAs bind to relatively key proteins to directly regulate autophagy. Both circMUC16 (Gan et al., 2020), and circDNMT1, which binds to TP53 proteins (Du et al., 2018), promote autophagy. CiRS-7 promotes autophagy by increasing the expression of BECN1 and LC3-II (Cai et al., 2020).

### 4.4 Noncoding RNAs and necroptosis

NcRNAs play crucial roles in regulating necroptosis by targeting specific proteins and forming competitive networks. While miRNAs and circRNAs have been less studied in this context, early research has provided valuable insights for future investigations. LncRNAs can modulate necroptosis through signaling pathways or act as ceRNAs to indirectly regulate necroptosis. TRINGS, a p53-inducible lncRNA, can interact with STRAP to block the STRAP-GSK3 β-NF-κB pathway and protect tumor cells from necroptosis when its level rises (Khan et al., 2017). The involvement of lncRNAs as ceRNAs in necroptosis is much more common. For example, lncRNA Linc00176 induces necroptosis in HCCs by forming a competitive network with genes such as miR-9 and miR-185, affecting the cell cycle and HCC survival (Tran et al., 2018). LncRNA H19-derived miR-675, promoted HCC necroptosis by targeting Fas-linked proteins with death structural domains, leading to elevated levels of p-MLKL and RIP3 as well as reduced expression of FAD (Harari-Steinfeld et al., 2021). LncRNA-NRF inhibits cardiomyocyte necroptosis by targeting miR-873 (Wang et al., 2016). The way miRNAs are involved in necroptosis is mainly by targeting associated proteins. For example, knockdown of miR-21 prevented the activation of hepatocyte necroptosis in mice by negatively regulating CDK2AP1 and could partially attenuate liver injury in cholestasis (Afonso et al., 2018). Upregulation of miRNA-223-3p reduced RIP3-mediated necroptosis by targeting RIP3 after spinal cord injury, thereby significantly alleviating spinal cord neuronal injury (Wang et al., 2019). MiRNA-223-3p plays a role in spinal cord injury, and is involved in ischemia/reperfusion (I/R). MiR-223-3p directly inhibits the expression of NLRP3 and IκB kinase α, two important mediators that can be involved in I/R-induced necroptosis. Whereas miR-223-5p can synergize with miR-223-3p, to ultimately inhibit cardiomyocyte necroptosis by targeting TNFR1 and DR6 (Qin et al., 2016). It has been demonstrated that in Parkinson’s disease (PD), miR-425 deficiency triggers necroptosis in dopaminergic neurons, and targeting miR-425 in 1-methyl-4-phenyl-1,2,3,6-tetrahydropyridine (MPTP)-treated mice restored dysfunctional dopaminergic neurodegeneration and ameliorated behavioral deficits (Hu et al., 2019). However, its specific mechanism is still not elucidated and needs to be studied in depth. Although there is less research on the topic of circRNAs and necroptosis, some preliminary findings have laid the groundwork for the development of this field. Gao et al. (Gao et al., 2022) found that circRNA CNEACR could directly bind to histone deacetylase (HDAC7) and interfere with its nuclear entry, which led to the attenuation of HDAC7-dependent repression of forkhead box protein A2 (Foxa2) transcription, which could repress the Ripk3 gene by binding to its promoter region. Ultimately, the inhibition of necroptosis in mouse cardiomyocytes significantly reduced myocardial infarct size and improved cardiac function.

### 4.5 Noncoding RNAs and pyroptosis

NcRNAs play a crucial role in regulating cellular pyroptosis, impacting disease pathophysiology. LncRNAs modulate pyroptosis by fine-tuning miRNA function and influencing inflammatory vesicle formation. The crosstalk between lncRNAs, miRNAs, and mRNAs forms a ceRNA network that regulates specific pyroptosis signaling cascades. LncRNA ADAMTS9-AS2 activates NLRP3 inflammatory vesicles and triggers cisplatin-treated cisplatin-resistant GC cells pyroptosis by targeting miR-223-3p (Ren et al., 2020). Similarly, lnc00958 was shown to promote cancer cell survival by downregulating miR-4306 levels to activate the absence in melanoma 2/Gasdermin D(GSDMD)-mediated pyroptosis pathway (Jiang et al., 2021). LncRNA NEAT1 regulated Ionizing radiation (IR)-triggered pyroptosis in colorectal cancer cells by affecting the miR-448/gasdermin E network (Su et al., 2021). The pathway by which lncRNAs regulate pyroptosis is manifested in the suppression of inflammatory vesicle expression. For instance, knockdown of lncRNA XIST increased the expression levels of NLRP3, cysteinyl asparagin-1, IL-1β, IL-18, and GSDMD-N and activated NSCLC cells to undergo pyroptosis (Xu et al., 2020). In ovarian cancer, it is manifested that knockdown of lncRNA GAS5 activates pyroptosis by suppressing the expression of apoptosis-associated speck (ASC), cysteine asparaginase-1, IL-1β and IL-18 (Li et al., 2018). MiRNAs is necessary in participating in pyroptosis as a member of the ncRNA family. MiRNAs can regulate pyroptosis both through specific signaling cascades and by mediating inflammatory vesicles or GSDM family proteins. MiR-181 induces pyroptosis by affecting the SIRT1/PGC-1a/Nrf2 signaling cascade reaction, which increases the levels of pyroptosis-associated proteins, such as NLRP3, cysteinyl asparagin-1, IL-1β and IL-18 (Zhao et al., 2019). Gu, Y. et al. found that both overexpression of miR-200b and reduction of JAZF1 can lead to activation of the NF-κB signaling pathway, which is a key player in coordinating pyroptosis (Gu et al., 2022). Thus, miR-200b can induce pyroptosis in breast cancer cells by regulating the JAZF1/NF-κB axis. MiRNAs also play a crucial role in the regulation of pyroptosis-related proteins as well as inflammatory vesicles. Elevated MiR-145 elevates the expression of GSDMD, IL-β, and IL-18, activates cysteinyl asparaginase-1-mediated pyroptosis, and may be useful in the treatment of cervical cancer (Yu et al., 2020). Cetuximab is commonly used in the treatment of triple-negative breast cancer (TNBC). Xu, W. et al. found that Cetuximab combined with the miR-155-5p antagomir could enhance the expression of GSDME-N and caspase-1, promoting pyroptosis in TNBC cells and making the organism less resistant to cetuximab (Xu et al., 2021b). Existing studies have shown that the mechanism by which circRNA regulates pyroptosis is the initiation of pyroptosis-related genes (Gao et al., 2021). On the one hand, circRNA initiates DNA methylation of pyroptosis-related genes. circRNA-0001836 is the activation of pyroptosis by promoting the expression of NLRP1 through DNA demethylation (Tan et al., 2021a). On the other hand, circRNA releases the inhibitory effect of miRNAs on downstream targets that act as miRNA sponges. For example, in lung adenocarcinoma, circNEIL3 achieves the effect of activating pyroptosis by directly binding to miR-1184 (Zhang et al., 2022). While ncRNAs’ role in PCD has been extensively studied, research on ncRNAs and PANoptosis is still in its early stages, requiring further exploration and attention from researchers.

## 5 Regulatory roles of noncoding RNAs-PCD axis in osteoporosis

**FIGURE 5:**
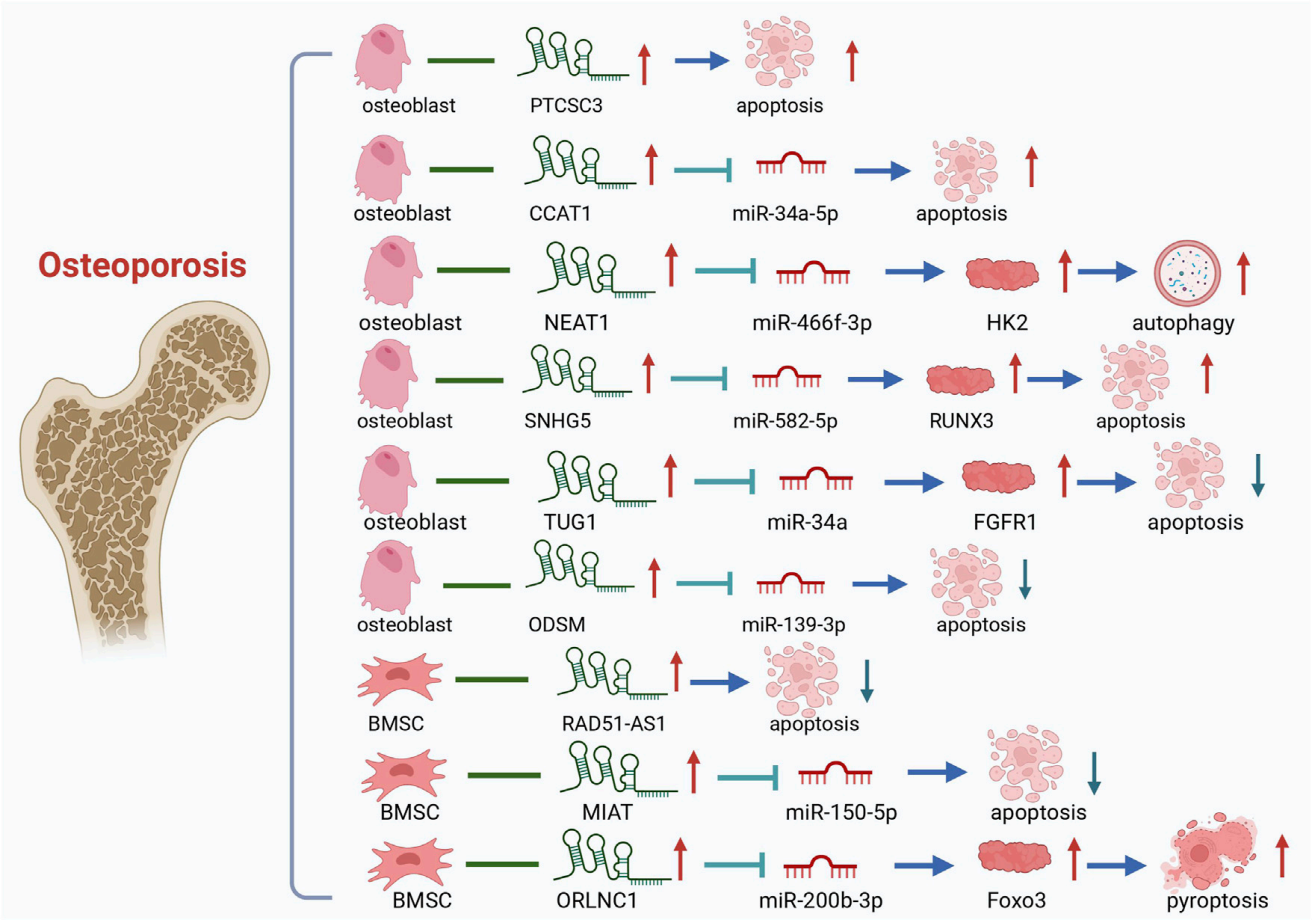
LncRNAs-PCD Axis in OP. It summarizes that lncRNAs regulate PCD in bone cells.

Recent research focuses on the ncRNAs-PCD axis and its role in disease regulation. Understanding its regulatory mechanisms is crucial for targeted therapy. Studies have highlighted its involvement in osteoporosis, particularly in regulating osteoblast and osteoclast functions (as shown in Figure 5).

Research on the role of lncRNAs in OP has shown that they can act as both promoters and inhibitors of osteogenic apoptosis. Elevated levels of certain lncRNAs accelerate OP progression, while others can be targeted for OP therapy to inhibit osteogenesis and alleviate the condition. LncRNA PTCSC3 has been verified to be significantly upregulated in patients with OP, and its upregulated level is positively correlated with the disease stage of OP. Overexpression of PTCSC3 promotes osteoblast apoptosis, whereas PTCSC3 silencing inhibits osteoblast apoptosis (Liu et al., 2022c). SNHG5 exerts its function in hBMSC osteogenesis and apoptosis by targeting the miR-582-5p/RUNX3 axis. Silencing of SNHG5 inhibited osteogenic differentiation and induced apoptosis in hBMSCs (Zheng et al., 2020a). TNF-α can inhibit the proliferation, ALP activity and mineralized nodule formation of mouse embryo osteoblast precursor cells (MC3T3-E1) cells, downregulate the expression of lncRNA TUG1 in MC1T3-E3, and promote apoptosis. And EGCG and TNF-α act antagonistically to each other, which can attenuate the inhibition of MC3T3-E1 cells by TNF-α, inhibit osteoblast apoptosis, and promote the progression of OP (Han et al., 2022). In the rat model, CCAT1 downregulated the expression of miR-34a-5p, which aggravated the pathological changes of the bone tissue of OP OVX rats and promoted osteoblast apoptosis. In OP cell models, lncRNA colon cancer-associated transcript-1 (CCAT1) was found to inhibit miR-34a-5p expression to inhibit differentiation, mineralization capacity, and proliferation, and to promote apoptosis of OVX rat osteoblasts *in vitro*. The relationship between CCAT1 and osteoblast apoptosis and OP was further verified (Hu et al., 2021). Experiments have shown that iron accumulation (IA) can lead to high expression of lncRNA XIST and promote osteoblast apoptosis through miR-758-3p/caspase 3, which in turn leads to OP (Liu et al., 2021a). The above are some promoters of osteogenic apoptosis, and the following are some inhibitors of osteogenic apoptosis, whose elevated levels are beneficial to bone formation. *In vitro* experiments showed that lncRNA ODSM inhibited osteoblast apoptosis (Wang et al., 2018a). Wang, Y. et al. conducted *in vivo* experiments in mice and found that lncRNA ODSM, which may be regulated in the MG unloading environment, partially reduced apoptosis and promoted the differentiation of MC3T3-E1 cells by interacting with miR-139-3p (Wang et al., 2020a). LncRNA RAD51-AS1 increases viability and osteogenic differentiation but decreases apoptosis of hBMSCs, a mechanism that may shed light on the treatment and prevention of OP (Li et al., 2023a). LncRNA MIAT regulates the proliferation, apoptosis, and osteogenic differentiation of BMSCs by targeting miR-150-5p. When MIAT was downregulated, the osteogenic differentiation of BMSCs was promoted, and apoptosis was inhibited, suggesting a potential role of MIAT in OP treatment (Wang et al., 2022a). LncRNA TUG1 interacted with miR-34a as ceRNA and upregulated FGFR1 protein expression. LncRNA TUG1 was upregulated in FSS-cultured osteoblasts, which promoted osteoblast proliferation and inhibited apoptosis (Wang et al., 2021d). Some LncRNAs have been shown to affect bone composition through autophagy and are associated with the progression of OP. The expression of lncRNA NEAT1 was upregulated in OP bone tissues and osteoblasts. Zhao, X. et al. found through bioinformatics analysis and diluciferase reporter gene assay that NEAT1 knockdown significantly inhibits autophagy in osteoblasts both *in vitro* and *in vivo*. Meanwhile, knockdown lncRNA NEAT1 inhibits osteoblast autophagy through the miR-466f-3p/HK2 signaling pathway to regulate autophagy and bone metabolism in OP (Zhao et al., 2022). LncRNA can also participate in pyroptosis and regulate OP. Zhang, L.et al. found that lncRNA ORLNC1 was associated with the progression of OP (Zhang et al., 2021b). As a ceRNA of miR-200b-3p, lncRNA ORLNC1 affected BMSCs through the miR-200b-3p/Foxo3 pathway to pyroptosis and promoted the development of OP (as shown in Table 1).

**TABLE 1:** LncRNAs-PCD axis in OP.

LncRNAs	Function	Mechanism	References
lnc-XIST	Promotes osteoblasts apoptosis	miR-758-3p/caspase 3	Liu et al. (2021a)
lnc-SNHG5	Promotes osteoblasts apoptosis	miR-582-5p	Zheng et al. (2020a)
lnc-000052	Promotes osteoblasts apoptosis	miR-96-5p-PIK3R1	Li et al. (2020b)
lnc-CCAT1	Promotes osteoblasts apoptosis	miR-34a-5p	Hu et al. (2021)
lnc-PVT1	Promotes osteoblasts apoptosis	miR-497-5p/HMGA2	Ji et al. (2021)
lnc-HOTAIR	Promotes osteoblasts apoptosis	miRNA-138	Xu et al. (2021a)
lnc-MALAT1	Inhibits osteoblastic apoptosis	miR-485-5p/WNT7B	Zhou et al. (2022b)
lnc-KCNQ1OT1	Inhibits osteoblastic apoptosis	miR-141-5p	Wang et al. (2021c)
lnc-AK077216	Inhibits osteoclast apoptosis	NIP1/RANKL/NFATc45	Liu et al. (2019)
lnc-TUG1	Inhibits osteoblastic apoptosis	TNF-α/MC3T3-E1	Han et al. (2022)
lnc-ODSM	Inhibits osteoblastic apoptosis	miR-139-3p	Wang et al. (2018b)
lnc-MIAT	Inhibits osteoblastic apoptosis	miR-150-5p	Wang et al. (2022a)
lnc-TUG1	Inhibits osteoblastic apoptosis	miR-34a/FGFR1	Wang et al. (2021d)
lnc-GAS5	Inhibits osteoclasts apoptosis	miR-21	Cong et al. (2020)
lnc-NEAT1	Promotes osteoblasts autophagy	miR-466f-3p/HK2	Zhao et al. (2022)
lnc-ORLNC1	Promotes BMSCs pyroptosis	miR-200b-3p/Foxo3	Zhang et al. (2021b)

Increasing high-throughput RNA sequencing and circRNAs microarray studies indicated that circRNAs are differentially expressed in OP (Chen et al., 2021c). The current study found that circRNAs can participate in the occurrence of OP by regulating apoptosis and autophagy in osteocytes. Zhou, R. et al. found that Circ_0000020 was upregulated during the osteogenic differentiation of BMSCs and regulated the BMP2/Smad pathway by sponging miR-142-5p as ceRNA to regulate the osteogenic differentiation of BMSCs (Zhou et al., 2021). Silencing Circ_0000020 can inhibit osteogenic differentiation, promote osteoblast apoptosis, and inhibit ALP activity and mineralization capacity. CircRNA can not only function individually, but also act as ceRNA of miRNA, jointly regulating bone metabolism.

The involvement of miRNAs in apoptosis has been most studied in bone formation and bone resorption. MiRNAs not only promote osteoblast apoptosis but also inhibit it. MiRNAs that promote osteoblast apoptosis, and their inhibitors may shed light on future studies of drugs for the treatment of OP. However, elevated levels of miRNAs that inhibit osteoblast apoptosis ameliorate OP and are equally important to study. Zhou, B. et al. found that miR-483-3p expression was reduced in bone tissue samples from OP patients (Zhou et al., 2020). However, in miR-483-3p overexpressed human osteoblasts, cell viability, DNA synthesis ability and osteogenic capacity were promoted, and apoptosis was inhibited. The target of miR-483-3p is expected to provide ideas for the treatment of OP. Zhang, B. et al. found that miR-181a regulates DUSP6 expression during osteoclast differentiation, which modulates ERK and SMAD2 signaling pathways (Zhang et al., 2021a). MiR-181a positively induces differentiation and inhibits apoptosis of osteoclasts through DUSP6, leading to the development of OP. MiRNA can function alone or form a competitive network, which together regulate PCD in osteoblasts, affect the expression of bone density, and promote or inhibit the development of OP. Lnc_000052 and PIK3R1 share a miRNA target, miR-96-5p, which is downregulated in OP BMSCs. The downregulation of miR-96-5p can inhibit the effect of LNC_000052 knockdown, whereas miR-96-5p upregulation and LNC_000052 knockdown improves therapeutic outcomes in BMSC (Li et al., 2020b). CircRNAs and miRNA networks have been shown to induce or inhibit OP by regulating apoptosis and affecting homeostasis of osteoblasts and osteoclasts. They may affect osteoporosis through the Wnt signaling pathway or the RANKL-RANK pathway. It may also indirectly improve OP by regulating factors like GCs and PTH (Huang et al., 2022). CircRNAs act as miRNA sponges by competing with mRNAs for miRNA response elements. In an experiment to investigate whether circ-Rtn4-modified BMSCs (Rtn4-Exos) could regulate osteoblast apoptosis by affecting TNF-α, the miR-146a/Rtn4-Exos network was found to inhibit TNF-α-induced cytotoxicity and apoptosis of mouse MC3T1-E1 cells, suggesting that Rtn4-Exos could serve as a novel drug for the treatment of OP (Cao et al., 2020).

MiRNA also regulates autophagy in osteoblasts. The lack of autophagy in osteoblasts leads to the accumulation of harmful substances within the cell, which eventually leads to apoptosis and disease progression. However, overactivation of autophagy can also lead to PCD, called “autophagy-dependent cell death” or autophagy (Samara et al., 2008). Therefore, understanding the mechanisms of autophagy is essential for regulating its activity. Certain miRNAs can promote autophagy in osteoblasts. Currently, miR-199a-3p was found to induce autophagy by targeting IGF-1 and mTOR, which exacerbates OP under estrogen-deficient conditions (Fu et al., 2018a). MiR-1252-5p promotes OP by activating autophagy and inhibiting osteoclastogenic viability, reversing it through the miR-1252-5p/GNB1 axis may be a potential therapeutic strategy for OP (Huang et al., 2021a). Lu et al. observed that miR-15b modulates KDM7B by targeting USP6, ultimately inhibiting osteoblast autophagy and aggravating OP (Lu et al., 2021). MiR-152-5p regulates osteogenic differentiation through ATG14-mediated autophagy, reducing endogenous ROS accumulation and maintaining cellular redox homeostasis, so regulating oxidative stress and therapeutic inhibition of miR-152-5p may be an effective anabolic strategy for OP (Li et al., 2022). Overall, even autophagy, which is often considered “protective,” inhibits osteoblast survival and negatively affects osteogenic differentiation. Therefore, modulating intracellular miRNAs to maintain appropriate levels of autophagy may be an ideal therapeutic strategy for the treatment of OP (As shown in Table 2).

**TABLE 2:** The regulatory effect of miRNAs on apoptosis and autophagy in osteoclast.

MiRNAs	Function	Mechanism	References
miR-133a	Promotes osteoblasts apoptosis	MAPK/ERK	Wang et al. (2021b)
miR-139-3p	Promotes osteoblasts apoptosis	lncRNA ODSM	Wang et al. (2020a)
miR-140-3p	Promotes osteoblasts apoptosis	PTEN/PI3K/AKT	Yin et al. (2020)
miR-223-3p	Promotes osteoblasts apoptosis	FGFR2	Wang et al. (2021a)
miR-425-5p	Promotes osteoblasts apoptosis	Annexin A2	Chen et al. (2021a)
miR-4739	Promotes osteoblasts apoptosis	ITGA10/PI3K	Song et al. (2022)
miR-26a	Inhibits osteoblastic apoptosis	EZH2	Li et al. (2020a)
miR-27a-3p	Inhibits osteoblastic apoptosis	CRY2/ERK1/2	Ren et al. (2021)
miR-96-5p	Inhibits osteoblastic apoptosis	Abca1	Wang et al. (2022c)
miR-107	Inhibits osteoblastic apoptosis	AMPK-Nrf2	Zhuang et al. (2020)
miR-150-3p	Inhibits osteoblastic apoptosis	Runx2/Ostrix	Qiu et al. (2021)
miR-206	Inhibits osteoblastic apoptosis	Elf3/ALP	Huang et al. (2021b)
miR-214	Inhibits osteoblastic apoptosis	circ_0001843/HNGF6A	Zhu et al. (2020)
miR-708	Inhibits osteoblastic apoptosis	MC3T3-E1/H2O2/PTEN	Zhang et al. (2020b)
miR-758-3p	Inhibits osteoblastic apoptosis	lncRNA XIST/caspase 3	Liu et al. (2021a)
miR-3175	Inhibits osteoblastic apoptosis	Nrf2/DDB1/DCAF1	Chen et al. (2021b)
miR-4523	Inhibits osteoblastic apoptosis	Nrf2/PGK1	Liang et al. (2021)
miR-497	Inhibits osteoblastic apoptosis	TGF-β1/Smads	Gu et al. (2020)
miR-199a-3p	Promotes osteoblastic autophagy	IGF-1/mTOR	Fu et al. (2018a)
miR-1252-5p	Promotes osteoblastic autophagy	GNB1	Huang et al. (2021a)
miR-15b	Inhibits osteoblastic autophagy	USP7/KDM6B	Lu et al. (2021)
miR-152-5p	Inhibits osteoblastic autophagy	ATG14/ROS	Li et al. (2022)
miR-466f-3p	Inhibits osteoblastic autophagy	lncRNA NEAT1/HK2	Zhao et al. (2022)

Understanding the pathways through which miRNAs regulate osteoblast pyroptosis is essential for OP research. MiR-200b-3p forms a competitive network with lncRNA ORLNC1, Foxo3 is the target of miR-200b-3p, and ORLNC1 promotes CML-induced pyroptosis of BMSCs by targeting the miR-200b-3p/Foxo3 pathway. Therefore, miR-200b-3p can inhibit the pyroptosis of BMSCs to inhibit the progression of OP (Zhang et al., 2021b).

## 6 Clinical applications of noncoding RNAs-mediated programmed cell death

Numerous studies are exploring the role of ncRNA-PCD in diseases like cancer and cardiovascular disorders, with potential applications in diagnosis and treatment. Ye, et al. summarize that curcumin treatment of autophagy and miRNA may be a promising mechanism and target for lung cancer treatment strategies (Ye et al., 2012). Zhang et al. identified miRNAs by high-throughput microarrays as a response to curcumin in human lung cancer (Zhang et al., 2010). Han et al. noted that lung cancer cells undergoing autophagy were resistant to EGFR-TKI and suggested that enhanced autophagy in the role in the poor performance of EGFR-TKI (Han et al., 2011). Although there is no evidence that curcumin works on both autophagy and miRNA, it can be understood as a new research direction to inform the study of drug mechanisms and targeted drug development. Sorafenib is a drug used to treat inoperable or distantly metastasis HCC, and Li et al. found that continuous sorafenib treatment reduced the expression level of circITCH in sorafenib-resistant HCC cells (Li et al., 2023b). The overexpression of circITCH is mediated by the regulation of miR-20b-5p/PTEN/PI3K/Akt signaling cascade response, increased sorafenib sensitivity, promoted apoptosis and decreased cell migration in sorafenib-resistant HCC cells. Resveratrol is a non-flavonoidal polyphenolic organic compound, and it has been demonstrated that resveratrol can be used to ameliorate PD symptoms by modulating the MALAT1/miR-129/SNCA pathway, which is involved in mitochondria-mediated apoptosis (Xia et al., 2019).

However, only a few studies have mentioned the clinical application of ncRNA-mediated PCD in orthopedic diseases. For example, artemisinin (ARS) inhibited osteoclast differentiation by down-regulating the RANKL-induced osteoclast formation pathway, which led to osteoclast ferroptosis, inhibited bone resorption, and improved OP (Zhang, 2020). However, ARS may lead to side effects such as hypocalcemia, so further studies are needed to evaluate the efficacy and safety of ARS for OP. Despite these advances, their application to clinical treatment remains an open question. However, as biomarkers of OP, changes in ncRNA expression may be detected in the early stages of the disease, which could help physicians to diagnose and intervene in a timely manner. In the development of OP drugs, certain specific ncRNAs and the peptides they encode may also be utilized as potential targets for intervention in OP, leading to the development of new therapeutic approaches. For example, precision gene therapy utilizing functional elements of lncRNA Nron can effectively inhibit the development of OP (Jin et al., 2021). Recently it has also been discovered that lipid nanoparticle and engineered extracellular vesicles-based RNA therapy can be used to treat OP. Liu et al. utilized bone-targeted lipid nanoparticle delivery of m7G-methylated Runx2 mRNA to promote bone formation in osteoblasts (Liu et al., 2024). Delivery of siRNA by engineered small extracellular vesicles can also be used to improve OP (Liu et al., 2023). In addition, doctors can monitor changes in ncRNA expression to assess the progression of patients' diseases, providing more possibilities for precision medicine in OP.

## 7 Conclusion

OP presents a significant global health challenge, and insights into bone metabolism are crucial for developing OP treatments. While the ncRNA-PCD axis has been studied in various diseases, including cancer and cardiovascular diseases, further research is needed to delve deeper into the mechanistic pathways and specific factors involved. The discovery of how ncRNAs and PCD regulate bone metabolism represents a major advancement in OP research. PCD plays a pivotal role in bone remodeling under both physiological and pathological conditions, with ncRNAs influencing OP development by regulating different types of PCD.

Some ncRNAs can either promote or inhibit osteoblast PCD, impacting bone formation and resorption, thus affecting OP progression. Targeting the ncRNA-PCD interaction could offer a promising avenue for novel OP treatments, potentially through the development of ncRNA-PCD mimics or inhibitors to prevent bone loss. By shedding light on the role of ncRNAs in interacting with PCD and their implications for OP, this study aims to inspire further research in the bone field to uncover deeper molecular mechanisms and potential treatment strategies for OP. However, more research is needed to explore the cellular and organismal effects of ncRNA-PCD interactions, including specific pathways in osteoblasts and osteoclasts, as well as potential side effects on these cells.
